# Adding a transversus abdominis plane block to parenteral opioid for postoperative analgesia following trans-abdominal hysterectomy in a low resource setting: a prospective, randomised, double blind, controlled study

**DOI:** 10.1186/s13104-016-1864-2

**Published:** 2016-01-28

**Authors:** Nomaqhawe Moyo, Farai D. Madzimbamuto, Samson Shumbairerwa

**Affiliations:** Department of Anaesthesia and Critical Care Medicine, University of Zimbabwe College of Health Sciences, Mazowe St, Harare, Zimbabwe

**Keywords:** TAP block, Trans abdominal hysterectomy, Postoperative pain

## Abstract

**Background:**

The current gold standard treatment for acute postoperative pain after major abdominal surgery is multimodal analgesia using patient controlled analgesia delivery systems. Patient controlled analgesia systems are expensive and their routine use in very low income countries is not practical. The use of ultrasound in anaesthesia has made some regional anaesthesia blocks technically easy and safe to perform. This study aimed to determine whether adding an ultrasound guided transversus abdominis plane block as an adjunct to the current parenteral opioid based regimen would result in superior pain relief after a trans abdominal hysterectomy compared to using parenteral opioids alone.

**Methods:**

Thirty-two elective patients having trans abdominal hysterectomy were recruited into a prospective randomised double-blind, controlled study comparing a bilateral transversus abdominis plane block using 21 ml of 0.25 % bupivacaine and 4.0 mg dexamethasone with a sham block containing 21 ml 0.9 % saline. Sixteen patients were allocated to each group. Anaesthesia and postoperative analgesia was left to the attending anaesthetist’s discretion. Primary outcome was visual analogue scale for pain at 2 h and 4 h. Secondary outcomes were time to first request for analgesia, visual analogue scale for comfort and bother. The data were analysed using the Statistical Package for Social Sciences (SPSS version 16).

**Results:**

There was no statistically significant difference in the demographics of the two groups regarding weight, height, physical status and type of surgical incision. There was a statistically significant difference in visual analogue scale for pain at 4 h during movement with lower pain scales in the test group (p = 0.034). Women in the control group had an average pain free period of 56.8 min (median 56.5 min) before requesting a rescue analgesic compared to 116.5 min (median 103 min) in the study group. The between group difference in the average total analgesia duration was statistically significant at the 0.05 level (p = 0.005).

**Conclusion:**

The addition of a bupivacaine–dexamethasone transverse abdominis plane block to intramuscular opioid does produce superior acute post-operative pain relief following a hysterectomy. However a single-shot block has a limited duration of action, and we recommend a repeat block.

*Trial registration*: *Clinical trials registration was obtained PACTR201501000965252.*http//www.pactr.org/ATMWeb/appmanager/atm/atmregistry?_nfpb=true&_windowLabel=BasicSearchUpdateController_1&BasicSearchUpdateController_1_actionOverride=%2Fpageflows%2Ftrial%2FbasicSearchUpdate%2FviewTrail&BasicSearchUpdateController_1id=965. *The trial was registered on the 12th Dec 2014*

## Background

Patients usually suffer significant pain after abdominal surgery, with the major source of pain being the anterior abdominal wall and the abdominal viscera [1]. The current gold standard acute postoperative analgesic regimen after major abdominal surgery is patient-controlled epidural analgesia (PCEA) or intravenous patient controlled analgesia (IVPCA) with a combination of narcotic and local anaesthetic drugs with or without intravenous non-steroidal anti-inflammatory drugs (NSAID) or paracetamol [2].

Postoperative analgesia at Harare Central Hospital (HCH) and Parirenyatwa group of hospital (PGH) in Zimbabwe consists of parenteral opioid administration with a general preference for intramuscular pethidine 3-h per rising need (prn), with morphine intramuscular being rarely used due to misconceptions that overestimate and overemphasise the risk of adverse effects and addiction [3]. With the use of traditional intramuscular injection of pethidine or morphine 30–50 % patients experience moderate to severe postoperative pain [4]. Patient controlled analgesia (PCA) pumps are expensive and their use in a very low income country is not practical especially with units that have a high patient volume, and are under-staffed. Besides being resource-consuming, PCA carries an infection risk and without close monitoring, places the patient at great risk of overdose and death [2].

A positive development is the advent of ultrasound use in anaesthesia, which has made a variety of regional anaesthesia blocks possible that may offer technically simple, safe and better alternative analgesic regimen or adjuncts [5]. A transversus abdominis plane TAP block has been used for a variety of abdominal surgery. A posterior TAP block can be used to provide postoperative analgesia for any lower abdominal surgery for example trans-abdominal hysterectomy, caesarean delivery, appendicectomy, urogynaecological procedures and colorectal surgery [6, 7]. The efficacy of a TAP block for analgesia is proven, its usefulness as an adjunct to various PCA modalities has been well studied. However, its use in the context of traditional intramuscular opioid use has not been reported. This is the context which is prevalent in low resource settings [8].

The aim of this study was to determine whether adding a regional anaesthesia technique, an ultrasound-guided TAP block, as an adjunct to the current parenteral opioid based regimen would result in superior pain relief after a trans-abdominal hysterectomy compared to the use of parenteral opioid alone.

## Methodology

This study was carried out at HCH and PGH. Ethical clearance to conduct the study was obtained from the Joint Parirenyatwa Hospital and College of Health Sciences Research Ethics Committee [JREC: 185/13] and the Medical Research Council of Zimbabwe [MRCZ/B/571] and Clinical trials registration was obtained from the Pan African clinical trials registry PACTR201501000965252. Data were collected between 20 September 2013 and 31 March 2014.

### Participants

The participating subjects were patients admitted at PGH and HCH for elective open trans-abdominal hysterectomy. The patients were American Society of Anesthesiologists (ASA) physical status grade I–III, aged between 18 and 60 years and gave consent. Exclusion criteria were as follows: history of local anaesthetic allergy, patients receiving drugs which could result in opioid tolerance, inability to understand study protocols, obesity BMI >35, weight <40 kg and patients who were unable or unwilling to give informed consent. Written informed consent was obtained in their language as well as an English translation made available from all participants.

### Design

**Fig. 1:**
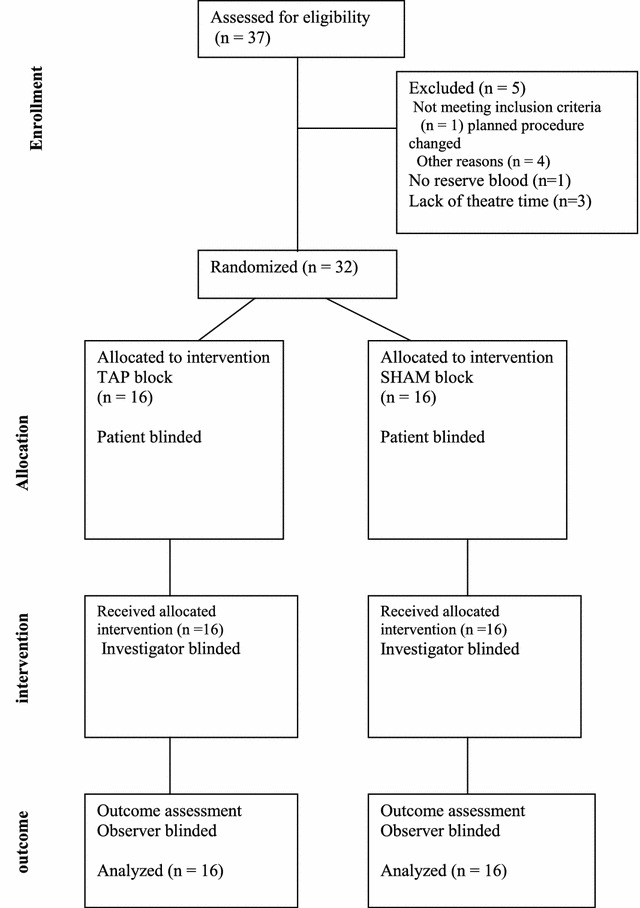
Recruitment flow chart

This was a prospective, randomised, double blind, controlled study. The hypothesis was that adding a Bupivacaine–Dexamethasone transversus abdominis plane (TAP) block to currently practised traditional im Pethidine 3 h (prn) as prescribed by the anaesthetist as part of a multimodal postoperative analgesic regimen produces superior postoperative pain relief compared to using Pethidine alone in patients who underwent TAH. A power calculation with an α value of 0.02 and a β value of 0.1 based on a difference in the proportion of patients with moderate to severe pain of 20 % with standard deviation of 15.2 % would require a sample size of 15 in each group. Including a non-response rate of 1 % a sample size of 32 patients (16 per group) was needed to obtain 90 % statistical power of the results. Thirty-seven patients were recruited, five were excluded from the study leaving 32 who were finally included. Three were excluded because they were postponed for lack of theatre time, and of the other two, one could not be done because blood was not reserved for surgery if needed. The remaining patient had the planned procedure changed. See Fig. 1.

Randomisation was carried out using sealed opaque envelopes with coupons labelled S for the control group and T for study group. The coupons were generated and sealed in envelopes before data collection was commenced. After obtaining written consent from the patient, she was asked to pick an envelope. An independent anaesthetic assistant prepared the injectate using clearly written instructions depending on the group the patient picked and supplied this to the investigator with the label ‘injectate’ written on it.

Sixteen patients were allocated into the control group [SHAM], they received a Sham TAP block using 21 ml 0.9 % normal saline on each side using the ultrasound guided posterior TAP block. The other 16 patients were allocated into the test group and received 20 ml 0.25 % bupivacaine plus 1 ml dexamethasone [4 mg] on each side. The TAP block was performed at the end of surgery before reversal of muscle relaxation and anaesthesia. Patients were blinded as to which group they had been allocated into. The investigator and staff involved in the assessment of the patient postoperatively noting the time to first analgesic request and assessment of the different visual analogue scale [VAS] were also blinded to which group the patient was allocated to.

All patients received a general anaesthetic as prescribed by the attending anaesthetist. An intravenous induction with either sodium thiopental or propofol, intubation with suxamethonium or atracurium, maintenance of anaesthesia with isoflurane, intravenous morphine analgesia and reversal with neostigmine and atropine was done. Standard monitoring used intraoperatively included electrocardiography [ECG], non-invasive blood pressure [NIBP], capnography, pulse oximetry and urine output. The prescribed postoperative analgesic regimen was intramuscular pethidine given 3 h prn and nurse controlled. To make the data collection convenient patients were kept in the recovery room until their first request for analgesia. NIBP, ECG and pulse oximetry were monitored in recovery.

The primary outcome measured was adequacy of postoperative pain relief, as assessed by a VAS for pain at 2 and at 4 h after surgery. Pain was assessed at rest and during standardised movement [knee flexion]. Secondary outcomes measured were: total analgesic duration, noted as the time from block application to the time to first rescue analgesic request by the patient, patient comfort as assessed using a VAS comfort and VAS bother which assessed if patients were bothered by their surgical wounds. Patients were instructed preoperatively on the use of the VAS.

The data were analysed using, the statistical package for social sciences (SPSS). Descriptive statistics were used to report measures of central tendencies for quantitative variables. Student’s *t* test for independent groups was used to test the hypothesis and also check relationships on continuous variables. Categorical variables were expressed as percentages and frequencies, and compared using the Chi square analysis. A p value <0.05 was considered to be statistically significant.

## Results

Table 1 summarises the demographic characteristics of the participants. There was no statistically significant difference in the demographics between the two groups with respect to weight, height, BMI, ASA physical status and type of surgical incision. There was a statistically significant in between- group difference in age, with the sham group being older. The age range in the sham group was 37–60 years compared to 30–52 years in the study group. All the patients recruited were analysed in their groups.

**Table 1:** Summarises the demographic characteristics of the participants

Period	TAP (n = 16)	SHAM (n = 16)	p value
Age (years, mean ± SD)	40.7 ± 6.8	46.5 ± 6.9	0.023
Weight (kg, mean ± SD)	67.3 ± 12.8	67.5 ± 11.3	0.968
Height (meters, mean ± SD)	1.7 ± 0.1	1.7 ± 0.1	0.751
Body mass Index (mean ± SD)	24.3 ± 3.8	24.3 ± 3.9	0.952
ASA physical status grading			
I [n (%)]	8 (50.0)	4 (25.0)	0.273
II [n (%)]	7 (43.8)	9 (56.3)	0.724
III [n (%)]	1 (6.3)	3 (18.8)	0.600
Type of incision			
Transverse [n (%)]	13 (81.3)	14 (87.5)	0.626
Longitudinal [n (%)]	3 (18.8)	2 (12.5)	0.626

Table 2 summarises VAS pain scores. VAS pain was assessed at rest and on movement at 2 and 4 h post intervention. There was no statistically significant difference in the mean VAS pain scores at rest between the TAP and SHAM group at 2 and 4 h post intervention, p value 0.087 and 0.853 respectively. The mean VAS pain score on movement at 2 h was 5.7 in the TAP group compared to 7.0 in the SHAM group. This difference was not statistically significant, (p value 0.09). Four hours post intervention the mean pain scores during movement were 4.8 in the TAP group compared to 6.2 in the SHAM group. This difference was statistically significant at 95 % level of testing (p value 0.034). This result means that dynamic pain at 4 h post TAP block was determined by the intervention.

**Table 2:** Summarises VAS pain scores

		Group TAP n = 16	Group SHAM n = 16	p value
VAS pain score at rest mean (median)	2 h post TAPB	4.1 [4]	5.3 (5.5)	0.087
VAS dynamic pain score mean (median)	2 h post TAPB	5.7 [6]	7.0 [8]	0.09
VAS pain score at rest mean (median)	4 h post TAPB	3.7 [4]	3.8 [4]	0.0853
VAS dynamic pain score mean (median)	4 h post TAPB	4.8 [4]	6.2 [6]	0.034

**Fig. 2:**
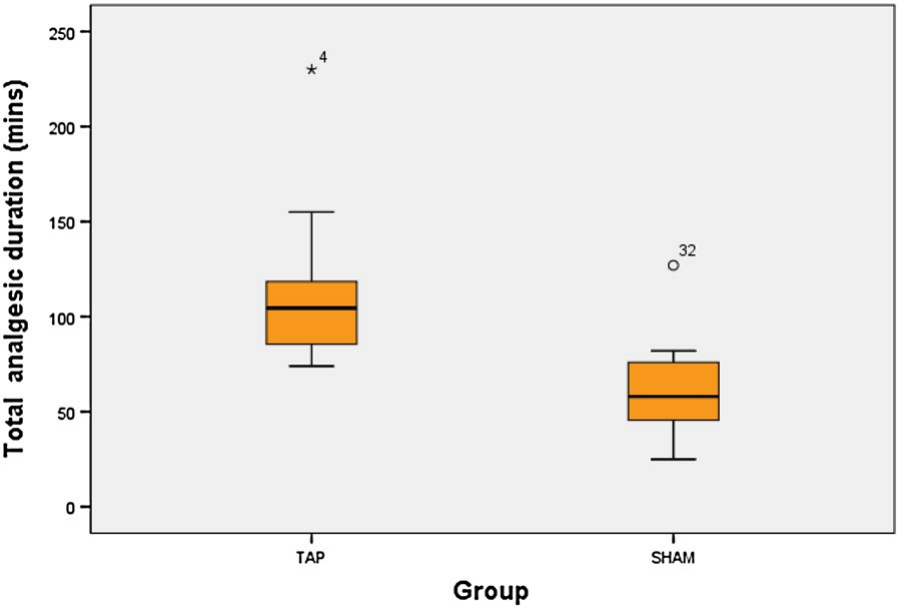
Total analgesic duration. Total analgesic duration was defined as the time period from TAP block performance to the time at first analgesic request

The box-and-whisker plot in Fig. 2 shows that 50 % of the patients in the TAP group requested their first rescue analgesic after 104 min post-intervention whereas 50 % of the patients in the SHAM group requested their first rescue analgesic within 57 min post-intervention. Total analgesic duration for the purpose of this study was defined as the time period from the TAP block performance to the time at first analgesic request. The findings show that women in the control group SHAM had an average pain free period of 56.8 min (median 56.5 min) before requesting for a rescue analgesic compared to the average of 116.5 min (median 103 min) in the study group TAP. The between-group difference in the average total analgesic duration is statistically significant (p < 0.005). This means that the total analgesic duration was determined by TAP intervention and on average those in the control group needed analgesic earlier as compared to those under TAP. The 95 % confidence interval for the difference in mean of TAP and SHAM intervention ranges from 25.7 to 93.7 min.

## Discussion

Shin et al. in their study, the pain scores during movement were superior in the TAP group at 4 h postoperatively compared to the control group. This implies that addition of a TAP block to the postoperative analgesic regimen improved dynamic pain relief [9]. Similarly in this study, pain score during movement at 4 h where lower in the test group compared to the control group. This is a positive finding as it supports early patient mobilisation, and generally improves patient cooperation with the enhanced recovery program activities [3]. Pain scores at rest were not statistically different between the two groups, probably because of the low pain levels normally experienced at rest [10].

This study was conducted to determine whether or not the addition of a bilateral ultrasound guided TAP block to our current postoperative analgesic regimen would provide superior pain relief in women undergoing elective TAH. We found that a TAP block intervention did improve pain relief in the immediate postoperative period as shown by the longer analgesic duration in the study group. The duration of analgesia in the study group was almost double that of the control group. Patients in the study group were pain free early postoperatively in the recovery unit, however there was no significant between-group difference in pain scores at rest or during movement at 2 h post intervention. This implies that a single shot TAP block has a limited duration of effect. The use of continuous catheter technique by infusion or intermittent injection of local anaesthetic into the TAP may be used to prolong the analgesia from of a block [5].

In a similar study by Marais et al. 2014 the researchers found that there was no statistically significant difference between the two groups in pain scores at rest or during movement. They attributed this to the use of intravenous opioid PCA as the primary postoperative analgesia, which is an effective analgesic regimen [11]. However, the addition of a TAP block reduced the total opioid consumption in the acute postoperative period in their study group [11].

Most studies reported on in the literature were performed in the setting of multimodal analgesic regimen using intravenous opioid, paracetamol or NSAID PCA, oral and per rectal paracetamol and NSAIDs as part of the enhanced recovery programs [3]. Intravenous paracetamol and NSAIDs are still quite expensive for routine use in resource limited settings. Addition of oral or per rectal paracetamol and NSAIDs to a multimodal analgesic regimen has an opioid sparing effect, reducing the total opioid consumption and opioid related side effects in the postoperative period [10]. At PGH and HCH addition of oral and per rectal formulations of paracetamol and NSAIDs to postoperative analgesic regimen could be feasible as they are low cost and readily available in the local market. It is thus a practical plan to have a multimodal analgesic regimen using ultrasound guided TAP block, oral or per rectal paracetamol and NSAIDs together with the traditional intramuscular pethidine in our resource limited setting. This approach would confer better pain relief and reduce the number of intramuscular opioid injections and accompanying concern about opioid related side effects. More research is required in this area. The major challenge with the introduction of oral analgesic drugs in our practice is the prolonged nil per oral intake orders in the postoperative period. Adoption of enhanced recovery programs in our practice may encourage early feeding of patients and thus enable the early introduction of oral analgesics. In a study by Marais et al. at the University of Cape Town, their multimodal analgesic regimen included the use of oral paracetamol 6 h and indomethacin 12 h per rectum in addition to the IV opioid PCA and TAP block [11].

Various drugs have been used as adjuvants for single shot regional anaesthetic blocks to improve quality and increase the duration of block. These drugs include clonidine, opioids, ketamine, neostigmine, adrenaline and glucocorticoids [12]. Regional blocks in which adjuvant drugs have been used include axillary, interscalene and supraclavicular brachial plexus blocks, sciatic nerve block, dental nerve blocks and TAP block [12, 13].

Ultrasound guided TAP block is easy to perform, with real time ultrasound images. There is usually an initial steep learning curve to the use of the ultrasound machine and performing the in plane needling techniques, but once these are mastered, a TAP block is one of the easy blocks to perform [14].

In the literature cases of procedure complication under ultrasound guidance are scanty. There is one report of liver trauma and peritoneal inflammation in a patient who had undergone inguinal hernia repair [9]. The other documented case by Farooq and Carey, the TAP block was performed using the blind landmark technique [15]. There are reports in the literature of toxic systemic levels of ropivacaine in a study where TAP block was done in women having open gynaecologic surgery. However, there were no reported clinical signs and symptoms of local anaesthetic toxicity in that study [16]. Recently Weiss et al., 2014 reported two case of convulsion post TAP block with ropivacaine [17]. In this study we did not measure serial plasma bupivacaine levels. There were no reported cases of procedure related complications. However this study was not powered to assess for TAP block safety.

The limitations of this study were the lack of strict control on the administration of the postoperative analgesia as it was nurse controlled. Bias could arise from the lack of standardization of the postoperative analgesia administration.

## Conclusion

This study investigated the utility of adding a regional anaesthetic technique, a TAP block as part of a prn opioid analgesic regimen in a low resource setting.

The study showed that compared to using traditional methods of prn opioid analgesia alone, the addition of an anaesthetist performed ultrasound-guided bupivacaine–dexamethasone transversus abdominis plane block as part of a multimodal analgesic regimen does produce superior acute postoperative pain relief following a TAH.

We recommend the routine use of a bupivacaine–dexamethasone TAP block as part of a wider multimodal analgesic regimen after a TAH. However, a single-shot TAP block has a limited duration of action; a repeat interval TAP block could prolong the analgesic effect in the ward. However this has not been tested in our study.

## Abbreviations

ASA: American Society of Anaesthesiologists
BMI: body mass index
ECG: electrocardiogram
HCH: Harare central hospital
i.m: intramuscular
IVPCA: intravenous patient controlled analgesia
JREC: Joint Parirenyatwa Hospital and College of Health Sciences Research Ethics Committee
MRCZ: Medical Research Council of Zimbabwe
NIBP: non invasive blood pressure
NSAID: non steroidal anti-inflammatory drug
PACTR: Pan African Clinical Trials Registry
PCA: patient controlled analgesia
PCEA: patient controlled epidural analgesia
PGH: Parirenyatwa group of hospitals
PRN: per rising need
SHAM: serious harm and morbidity
SPSS: statistical package for social sciences
TAH: trans abdominal hysterectomy
TAP: transversus abdominis plane
VAS: visual analogue scale
